# Pressurized Liquid Extraction of Bioactive Compounds from Seeds and Sprouts *Trigonella foenum-graecum* L. (Fenugreek): Enhanced Antioxidant and Anti-Hyperglycemic Activities

**DOI:** 10.3390/foods14122021

**Published:** 2025-06-07

**Authors:** Geovanni Silva Comilo, Karen Keli Barbosa Abrantes, Karina Miyuki Retamiro, Oscar de Oliveira Santos Junior, Wardleison Martins Moreira, Willyan Machado Giufrida, Celso Vataru Nakamura, Carlos Eduardo Barão, Lisiane dos Santos Freitas, Camila da Silva, Lucio Cardozo-Filho

**Affiliations:** 1 Postgraduate Program in Chemical Engineering, State University of Maringá, Av. Colombo, 5790, Maringá 87020-900, PR, Brazil; pg404458@uem.br (G.S.C.); oliveirasantos.oscardeoliveira@gmail.com (O.d.O.S.J.); wmmoreira@uem.br (W.M.M.); willyanmachado@gmail.com (W.M.G.); csilva@uem.br (C.d.S.); 2 Postgraduate Program in Agronomy, State University of Maringá, Av. Colombo, 5790, Bloco D-90, Maringá 87020-900, PR, Brazil; kkelibarbosa@gmail.com (K.K.B.A.); carlos.barao@ifpr.edu.br (C.E.B.); 3 Department of Basic Health Sciences, State University of Maringá, Av. Colombo, 5790, Bloco B-08 Sala 06, Maringá 87020-900, PR, Brazil; kmretamiro@uem.br (K.M.R.); cvnakmura@gmail.com (C.V.N.); 4 Federal Institute of Paraná (IFPR), Rua José Felipe Tequinha, 1400, Paranavaí 87703-536, PR, Brazil; 5 Department of Chemistry, Federal University of Sergipe, Av. Marcelo Deda Chagas, s/n, Bairro Rosa Elze, São Cristóvão 49107-230, SE, Brazil; lisiane.freitas@ufs.br

**Keywords:** pressurized *n*-propane extraction, *Trigonella foenum-graecum* L. (fenugreek), phytosterols, antioxidant, anti-tumor, anti-hyperglycemic properties

## Abstract

The present study examined the impact of germination using *Aloe vera* as an elicitor on the phytochemical composition, antioxidant capacity, and in vitro anti-hyperglycemic and antitumoral activity of fenugreek seed extracts germinated by pressurized *n*-propane. The lipid composition, free fatty acids, antioxidant activity, and phenolic content, as well as the contents of α-tocopherol, β-carotene, and minor compounds, have been determined for the extracts. The in vitro anti-hyperglycemic and anticancer activities were also evaluated in cervical cancer (HeLa) and colon cancer (SiHa) cell lines. Antioxidant activity increased two-fold, α-tocopherol increased almost three-fold, and β-carotene content was 55% higher in the germinated seed extracts compared to the raw. Fifteen polyphenolic compounds have been identified in fenugreek seed extracts, which promote germination by increasing high levels of polyunsaturated fatty acids at the expense of reducing saturated fatty acids. Extracts obtained from seed germination and elicitation with *Aloe vera* demonstrated potential in vitro anticancer activity in HeLa and SiHa cells. Fenugreek extracts demonstrated high in vitro inhibition of α-glucosidase (99%) and α-amylase (95%), indicating anti-hyperglycemic potential. The use of *Aloe vera* germination, combined with extraction using pressurized *n*-propane, demonstrated efficiency in enriching fenugreek seed extracts with bioactive compounds with potential in vitro anti-hyperglycemic and antitumor activity.

## 1. Introduction

*Trigonella foenum-graecum* L. (fenugreek), a member of the Fabaceae family, is widely cultivated in Asia, Africa, and the Mediterranean region. These seeds have bioactive compounds, including galactomannan, diosgenin, flavonoids, and phenolic acids, which contribute to a broad spectrum of pharmacological activities, such as anti-inflammatory, antimicrobial, cardiotonic, hepatoprotective, and lipid-lowering effects [1,2]. Germination has been recommended as a low-cost, easy-to-apply, and safe method for extracting and improving the bioactive compound profile of oilseeds. In the process of germination, the nutritional and medicinal values of seeds can be altered [3]. Nutrients such as vitamins, minerals, and limiting amino acids increase during germination [4].

Germinated seeds of *Sinapis arvensis* L. (wild mustard) showed an increase in antioxidant activity of up to 21 times [5]. Germination increased the content of vitamin E isomers in edible sprout seeds of *Fagopyrum tataricum* (tatar buckwheat) [6] and *Triticum aestivum* (wheat) [3] by two and 5.3 times, respectively. Germinating fenugreek seeds improved the nutritional profile and reduced the deficiency in fiber content, thereby enhancing its absorption by the body [7]. An increase in antioxidant activity and ascorbic acid content was also observed [8] in germinated fenugreek seeds.

*Aloe arborescens* Mill (*Aloe vera*) is known to have numerous bioactive properties that can contribute to improving the germination and phytochemical enrichment of fenugreek seeds. However, there is still a significant gap in research, and the potential of *Aloe vera* gel as a natural inducer of fenugreek germination has not yet been investigated. The polyphenols derived from *Aloe vera* may have various antioxidant properties [9] and consequently improve the profile of extracts obtained from seeds germinated in this solution.

Recent advances in extraction technologies have significantly improved the recovery and bioavailability of bioactive compounds from fenugreek seeds, expanding their possible applications. Ultrasound-assisted extraction using deep eutectic solvents (DES) [10] has shown great potential for improving the recovery of bioactive compounds [11]. Similarly, ultrasound-assisted extraction employing a binary solvent system (ethanol: *n*-hexane) has demonstrated greater extraction efficiency compared to traditional methods [9,10].

Other advances have focused on isolating bioactive compounds using supercritical CO_2_ extraction (sc-CO_2_), a scalable and solvent-free technique. The highest extract yield (2.57%) was obtained at 27.0 MPa and 42.6 °C, with molar solubility analyses confirming the key role of pressure and temperature in extraction efficiency. Compared to the hexane extract, extraction with sc-CO_2_ further optimized the recovery of steroidal sapogenin, yielding diosgenin, protodioscin, and sarsapogenin [12,13]. These findings highlight the effectiveness of sc-CO_2_ extraction in isolating high-value bioactives.

The developed study focuses on optimizing the bioactive extraction of compounds from raw and germinated *Trigonella foenum-graecum* (fenugreek) seeds using pressurized fluid extraction (PFE) technology. While conventional methods often compromise thermally labile compounds or require extensive post-processing, PFE operates at reduced temperatures to preserve heat-sensitive molecules (e.g., sterols and vitamins) and minimize contamination risks [14]. The present investigation selected pressurized *n*-propane as the solvent due to its high solubility in vegetable matrices, rapid processing times, low solvent consumption, and absence of residual contaminants [15]. This study aimed to evaluate the effect of pretreating germinating fenugreek seeds, using *Aloe vera* as an elicitor, on the phytochemical composition, in vitro antioxidant capacity, anti-hyperglycemic activity, and in vitro potential anticancer activity of extracts of fenugreek seeds germinated by pressurized *n*-propane. By integrating sustainable extraction techniques with advanced food processing strategies, this work advances the development of functional foods and pharmaceutical products based on fenugreek, offering scalable solutions for health promotion applications.

## 2. Materials and Methods

### 2.1. Materials

The fenugreek seeds (FGS) used in this experiment were sourced from a store specializing in natural products in Maringa, Paraná. The *Aloe Arborescens* Mill. was obtained from plants cultivated in the medicinal garden of the Insect Botanical Museum (MUBI) at the State University of Maringa, PR. *n*-Propane (Linde, São Paulo, SP, Brazil, 95% purity) was used as a solvent for extractions using pressurized fluids. The chemicals ABTS^•+^ [2,2-azino-bis-(3-ethylbenzthiazoline-6-sulfonic acid)] (Sigma-Aldrich, ≥98% purity) and Trolox (6-hydroxy-2,5,7,8-tetramethylchroman-2-carboxylic acid, Sigma-Aldrich, 97% purity) were used for the antioxidant analyses. HeLa and SiHa cells were provided by Dr. Luiza L. Villa (ICESP, School of Medicine, University of São Paulo/Brazil) and Dr. Silvya S. Maria-Engler (Faculty of Pharmaceutical Sciences, University of São Paulo/Brazil).

### 2.2. Raw Fenugreek Seeds

The raw seeds were washed with distilled water to remove potential pathogens. The samples were then dried in an oven at a controlled temperature of 318.0 K for 8 h, reaching a relative humidity (RH) of between 9% and 12%.

### 2.3. Determination of the Concentration of Aloe Vera Gel and Germination Time

Two solutions containing 20% and 30% *Aloe vera* gel were tested in Petri dishes containing approximately 200 seeds per dish for 48 h of germination at room temperature (24° C) in the dark. As there was no significant difference in the germination rate between the solutions, the tests were carried out with the 30% solution of *Aloe vera* gel to increase the potential for incorporating bioactive compounds. Germination time was determined by visual analysis, starting from the emergence of the radicle. The seed mixture ratio was based on the methodology proposed by [16]. For the fenugreek seeds (raw and germinated), considering the volume occupied by the sprouts in the extractor and the laboratory’s research experience, it was decided to test more than one seed mixture ratio. Thus, there are extracts with a higher proportion of germinated seeds compared to raw seeds (FGS/GW 1:2 and FGS/GA 1:2), considering the two germination substrates (*Aloe vera* and water), and extracts with a higher proportion of raw seeds compared to germinated seeds (FGS/GW 2:1 and FGS/GA 2:1).

### 2.4. Germinated Fenugreek Seeds

Raw fenugreek seeds were sterilized with a 1% sodium hypochlorite solution for 15 min and washed with water. The seeds were then soaked in deionized water for 24 h to break dormancy, followed by a thorough rinse with deionized water. The seeds were germinated in two substrates: water and *Aloe vera* solution (AVS). The AVS used to germinate the seeds consisted of 70% *Aloe vera* gel and 30% deionized water. The seeds germinated in water were hydrated with distilled water in a ratio of 1:0.5 (seeds to water), and those germinated in AVS were hydrated with AVS in a ratio of 1:0.25 (seeds to AVS). Both groups were placed in plastic trays in a dark place for 96 h at a temperature of 24° C to promote germination. The process of wetting the seed began with the uniform spraying of 25% distilled water to be absorbed by the seed. The seeds were sprayed every 12 h as necessary, ensuring that the seed absorbed water but that no excess water was deposited on the surface of the filter paper.

After germination, the sprouts were dried in an oven at 308.0 K for 8 h. Both raw and germinated fenugreek seeds were ground separately using a Philips Walita PowerChop multiprocessor (1000 W, model RI7303) and then sieved through a Tyler series mesh (0.55 mesh) to ensure uniform particle sizes. Preliminary germination and concentration tests were carried out on the *Aloe vera* gel to determine the optimum germination and gel dilution conditions. Fenugreek extracts were obtained from raw fenugreek seeds (FGS), seeds germinated with *Aloe vera* gel solution (FGGA), seeds germinated in water (FGGW), and mixtures of seeds germinated in different substrates (FGS/GW and FGS/GA) in varying proportions (1:2 and 2:1).

### 2.5. Pressurized Liquid n-Propane Extraction

Extractions with *n*-propane were performed in duplicate in the same experimental apparatus described by Trentini et al. 2017 [16]. The extraction procedures cover FGS, FGGW, FGGA, and mixtures of germinated seeds in different substrates (FGS/GW and FGS/GA) at varying proportions (1:2 and 2:1). In extractions on a structured bed, to obtain FGS/GA or FGS/GW, the raw fenugreek seeds were added first, followed by fenugreek seeds germinated in water or *Aloe Vera* in 1: 2 (*w/w*) and 2:1 (*w/w*) ratios. Each sample weighs 90 g. The equipment consists of an extraction vessel with a length of 36 cm, an internal diameter of 2.3 cm, and an internal volume of 150 mL.

When pressurized, propane first interacts with the seeds, extracting the extract, which in turn acts as a co-solvent in this process, extracting the compounds of interest from the germinated fenugreek seeds, in accordance with the Jaski et al. 2022 [15]. The experiments were conducted at a temperature and pressure of 333 K and 4 MPa, respectively. At 333 K and 4 MPa, liquid propane (ρ ≈ 0.44 g cm^−3^) efficiently solubilizes neutral lipids, allowing extracts with ~69% polyunsaturated acids (PUFAs), > 60% phytosterols (β-sitosterol + campesterol), and a high retention of α-tocopherol (see Table 2). Higher temperatures or lower pressures reduced this selectivity and degraded tocopherols, confirming 333 K and 4 MPa as the optimum conditions for maximizing the yield and stability of bioactives. Higher pressures would add operational complexity with negligible technological improvement since 4 MPa offers sufficient density to dissolve target lipids while maintaining moderate polarity and high selectivity. The time taken for extraction was 40 min at a constant flow rate of 2.0 mL min^−1^.

The *n*-propane was pumped and pressurized using a Teledyne ISCO 500D syringe pump into an extraction container filled with the samples. The entire extraction procedure was conducted in the absence of light. After 40 min, the extract was collected in an amber glass vial. The percentage extraction yield was calculated on a dry basis, as shown in Equations (1) and (2) below:

(1) $$\%\;R=\frac{M_{extracted}}{M_{dry}}\;x\;100$$

(2) $$M_{dry}=M_{FGS}+M_{FGG}-\left(M_{FGS}+M_{FGG}\right)*RH$$

where *M_FGS_* is the fenugreek seed mass; *M_FGG_* is the germinated fenugreek seed mass; and *RH* is the relative humidity.

### 2.6. Characterization of the Extracts

#### 2.6.1. Antioxidant Activity (ABTS^•+^ Method)

Antioxidant capacity is assessed using the ABTS^•+^ method, with Trolox (6-hydroxy-2,5,7,8-tetramethylchroman-2-carboxylic acid) as the reference antioxidant [17]. Samples (30 mL) of FGS, FGGW, FGGA, and mixtures were prepared and pipetted into test tubes. Then, 3 mL of ABTS^•+^ solution was added to the test tubes and homogenized. After 6 min in the dark, absorbance at 734 nm was measured on a spectrophotometer (Hach/DR 2800). The results were calculated from the Trolox calibration curve equation and expressed in mmol of Trolox per g of extract. All samples were analyzed in duplicate and kept shielded from light to prevent photodegradation.

#### 2.6.2. Determination of Total Phenolic Compounds (TPCs)

A total of 500 mg of extract was dissolved in 1.5 mL of *n*-hexane and then extracted with methanol (3 × 1 mL with stirring for 2 min), and the mixture was incubated for 16 h in the dark [18]. The methanolic extract was used to quantify the total phenolic compounds (TPCs). TPCs were quantified using a standard curve of gallic acid (EAG) and expressed in milligrams per gram of dry extract. The samples were analyzed in triplicate. The tests were conducted while ensuring protection from light.

#### 2.6.3. UHPLC-ESI-MS Profile of Phenolic Compounds

The detection of phenolic compounds was carried out using an ultra-high-performance liquid chromatograph (UHPLC) coupled to a triple quadrupole mass spectrometer (MS/MS), model Waters ZTQD LCMS Acquity, equipped with an electrospray ionization (ESI) source operating in positive ionization mode. Data were acquired and analyzed using MassLynx software version number 4.1, and the Pubchem database was used to identify compounds. Multiple reaction monitoring (MRM) mode was used to determine the analytes. The samples were subjected to a flow rate of 20 µL·min^−1^ and diluted in a ratio of 100 µL of the sample to 900 µL of HPLC-grade methanol. A 0.1% formic acid solution was added to the mixture. Operating conditions included a capillary voltage of 3.0 kV, a cone of 30 V, a desolvation temperature of 400 °C, and a nebulizer gas (nitrogen) flow of 600 L·min^−1^.

#### 2.6.4. β-Carotene Content

The β-carotene concentration is determined by dissolving the sample in *n*-hexane (1 mg·mL^−1^), reading the absorbance of the prepared solutions at 450 nm (Shimadzu, UV-1900), and calculating the β-carotene content in mg per 100 g of extract as described by Ogawa et al. [19]. All samples were analyzed in duplicate and kept shielded from light to prevent photodegradation.

#### 2.6.5. GC–MS Analysis

The fenugreek seed extract samples were derivatized with bis(trimethylsilyl) trifluoroacetamide (BSTFA) and trimethylchlorosilane to produce volatile trimethylsilylester derivatives. The sample was dissolved in dichloromethane and derivatized with 40 µL of BSTFA and 40 µL of pyridine. The solution was mixed in a vortex and left to stand for 30 min at 60 °C, after completing the volume to 1 mL with dichloromethane [20]. Derivatized samples were analyzed using GC/MS (Shimadzu QP2010 Plus, Shimadzu, Tokyo, Japan) equipped with a ZB-5MS column (60 m × 0.25 mm internal diameter (i.d.) × 0.25 μm thick film) for separation. An isothermal oven program was initially set at 90 °C for 2 min, increased to 160 °C at a rate of 2 °C min^−1^, and subsequently set as the final temperature at 280 °C and a rate of 15 °C min^−1^ for 10 min.

The injection was performed in split mode (1:20) at injector and detector temperatures of 280 °C and 300 °C, respectively. The carrier gas flow (He, ultra-pure, White Martins S.A., Aracaju, Brazil) was fixed at 1.0 mL min^−1^. The chemical components were tentatively identified, comparing the retention time and the main mass fragments at detectable peaks with those of authentic standards from the NIST-05 mass spectral library. The quantification analysis was performed considering the relative percentage area according to the equation: % A = (Peak area/Total area) × 100. All conditions were based on results obtained in previous studies [21].

### 2.7. In Vitro Evaluation of Cancer Cell Lines and Cell Viability Evaluation Using the MTT Colorimetric Method

The cytotoxicity of germinated fenugreek extracts (FGS/GA 2:1 and FGS/GA 1:2) was tested against SiHa and Hela. The cells were maintained at 37 °C in 5% CO_2_ and cultured in Dulbecco’s Modified Eagle’s Medium (DMEM) containing 10% fetal bovine serum (FBS) with the concentration adjusted to 2.5 × 10⁵ cells/mL. The extracts were applied at concentrations of 1000, 500, 100, and 50 μg·mL^−1^ directly on the cell monolayer, followed by incubation for 24 h at 310 K in a 5% CO_2_ atmosphere. After the incubation period, cytotoxic activity measurements were performed using a microplate reader (BIO-TEK Power Wave XS) set to a wavelength of 570 nm [22]. The samples were analyzed in triplicate.

### 2.8. Enzyme Inhibition Analysis of α-Glucosidase and α-Amylase

The procedure for assessing the inhibition of α-amylase and α-glucosidase enzymes followed the protocol described by Ayyash et al. (2018) [23]. For the α-amylase activity analysis, 100 μL of human saliva α-amylase (1.0 unit·mL^−1^) was mixed with 100 μL of the sample in phosphate buffer at pH 6.8 and incubated at 310 K for 5 min. A 250 μL starch solution (1 g·100 g^−1^) was added to initiate the reaction. The reaction was interrupted by adding 200 μL of DNS reagent, composed of 1% 3,5-dinitro salicylic acid and 12% potassium sodium tartrate in 0.4 mol·L^−1^ NaOH solution. The samples were heated to 373 K for 15 min, cooled in an ice bath, and diluted with 2 mL of distilled water. The absorbance was measured using a spectrophotometer set to 540 nm.

For α-glucosidase activity, 1 U·mL^−1^ of α-glucosidase (Sigma G5003, St. Louis, MO, USA) was dissolved in 0.1 mol·L^−1^ potassium phosphate buffer and adjusted to pH 6.8. The mixture was incubated with the sample at 310 K for 10 min. A 4-nitrophenyl α-D-glucopyranoside (Sigma N1377) solution was added to initiate the reaction, which continued for 30 min under the same temperature conditions. The reaction was stopped with 0.1 mol·L^−1^ sodium carbonate adjusted to pH 11.0. The absorbance was then measured using a spectrophotometer set to 400 nm. The samples were analyzed in triplicate.

### 2.9. Statistical Analysis

Analysis of variance (ANOVA), followed by Tukey’s test, was performed in the SISVAR statistical software, version 5.7, ensuring the reliability and accuracy of the experimental results [24]. The multivariate method of principal component analysis (PCA) was used to reduce the number of variables and evaluate their contribution to yield, ABTS^•+^, TPC, α-tocopherol, phytosterols, hydrocarbons, esters, fatty acids, and β-carotene for the FGS, FGGW, FGGA, and mixtures, using the computational package FactoMineR (R Studio Inc., Boston, MA, USA) [25].

## 3. Results and Discussion

### 3.1. Pressurized Liquid Propane Extraction

Table 1 shows the mass percentage yields of the extractions using different fenugreek seeds (FGS, FGGW, and FGGA), mixtures (FGS/GA and FGS/GW), and ratios (1:2 and 2:1). The data are not significant according to ANOVA, showing that there are no differences in the extraction yield with pressurized *n*-propane of the extracts obtained from raw and germinated fenugreek seeds, isolated or in mixture, and in different proportions (*p* < 0.05). The yield of raw, germinated fenugreek seed extracts and mixtures extracted by pressurized *n*-propane ranged from 3.1 to 4.6%. The yield of fenugreek seed extract extracted with *n*-hexane by Soxhlet at 65–70 °C for three hours was 5.5% [26]. Similar results were found by Jaski et al. 2022 [15] in a simultaneous extraction of sunflower seeds and olive leaves.

Although the yields were higher in the extraction by Soxhlet, the extraction time was much longer (180 min) compared to the extractions with *n*-propane (40 min), which would lead to increased production costs. Another factor is related to the lipid profile and the phenolic compound content of the extracts extracted with *n*-propane, from raw and germinated seeds, and from the mixtures, which were much higher (9 to 12 mg·100 g^−1^ of extract) compared to those obtained via Soxhlet (3.9 mg·100 g^−1^ of extract) [26].

### 3.2. Chemical Composition of Fenugreek Seed Extracts

#### 3.2.1. Content of Fatty Acids and Bioactive Compounds in Fenugreek Seed Extracts

Table 2 shows the fatty acids (FAs) and bioactive compounds in all fenugreek extracts extracted using pressurized *n*-propane.

**Table 2:** Composition of fatty acids and bioactive compounds present in raw fenugreek seed extract (FGS), germinated fenugreek seed extracts (FGGW and FGGA), and mixed extracts (FGS/GW and FGS/GA) in different proportions (1:2 and 2:1), obtained by pressurized *n*-propane.

Fatty Acids (%)	FGGW				FGGA				FGS/GW 2:1				FGS/GW 1:2				FGS/GA 2:1				FGS/GA 1:2				FGS			
Palmitic acid	7.8	±	0.1	^d^	8.3	±	0.1	^cd^	9.8	±	0.1	^bc^	9.2	±	0.2	^bc^	9.2	±	0.0	^ab^	10.8	±	0.7	^a^	11.0	±	0.2	^a^
Stearic acid	5.2	±	0.0	^b^	5.4	±	0.1	^ab^	5.2	±	0.0	^b^	5.1	±	0.0	^b^	5.2	±	0.1	^b^	5.3	±	0.0	^b^	5.6	±	0.1	^a^
Oleic acid	14.8	±	0.2	^b^	14.8	±	0.4	^b^	15.4	±	0.0	^b^	15.1	±	0.0	^b^	15.1	±	0.0	^b^	15.4	±	0.1	^b^	17.4	±	0.3	^a^
Linoleic acid	43.8	±	0.0	^a^	43.7	±	0.2	^a^	41.5	±	0.2	^bc^	42.6	±	0.1	^ab^	43.1	±	0.1	^a^	41.4	±	0.3	^bc^	40.5	±	0.9	^c^
Linolenic acid	25.3	±	0.2	^ab^	24.9	±	0.2	^b^	25.8	±	0.3	^c^	25.3	±	0.1	^ab^	24.7	±	0.1	^b^	24.5	±	0.2	^b^	23.0	±	0.3	^c^
Heneicosanoic acid	1.9	±	0.1	^a^	1.7	±	0.3	^a^	1.7	±	0.0	^a^	1.7	±	0.0	^a^	1.7	±	0.1	^a^	1.7	±	0.0	^a^	1.8	±	0.0	^a^
Behenic acid	1.2	±	0.2	^a^	1.3	±	0.4	^a^	0.7	±	0.0	^a^	1.1	±	0.0	^a^	1.0	±	0.0	^a^	1.0	±	0.0	^a^	0.7	±	0.0	^a^
MUFA^1^	14.8	±	0.2	^b^	14.8	±	0.4	^b^	15.4	±	0.0	^b^	15.1	±	0.0	^b^	15.1	±	0.0	^b^	15.4	±	0.1	^b^	17.4	±	0.3	^a^
PUFA^2^	69.1	±	0.2	^a^	68.6	±	0.1	^ab^	67.2	±	0.0	^c^	67.8	±	0.2	^bc^	67.8	±	0.2	^bc^	65.9	±	0.5	^d^	63.5	±	0.5	^e^
SFA^3^	16.1	±	0.0	^b^	16.6	±	0.4	^b^	17.3	±	0.1	^b^	17.1	±	0.2	^b^	17.1	±	0.2	^b^	18.7	±	0.7	^a^	19.1	±	0.3	^a^
**Bioactive compounds (mg 100 g^−1^ Extract)**																												
Squalene	146.7	±	5.6	^a^	132.0	±	3.4	^ab^	98.8	±	8.1	^b^	101.7	±	15.6	^c^	114.5	±	4.2	^bc^	88.4	±	1.7	^c^	102.8	±	4.5	^bc^
α-tocopherol	287.7	±	11.7	^a^	296.0	±	4.9	^a^	244.6	±	2.4	^b^	271.8	±	12.6	^ab^	273.3	±	3.5	^ab^	253.1	±	19.5	^ab^	99.4	±	18.3	^c^
CampEsterol	109.8	±	6.4	^ab^	92.4	±	3.4	^b^	144.7	±	13.5	^a^	93.9	±	1.2	^b^	131.9	±	18.5	^ab^	110.8	±	1.3	^ab^	114.5	±	12.1	^ab^
β-Sitosterol	683.3	±	5.5	^a^	660.9	±	11.5	^bc^	17.2	±	17.2	^b^	624.4	±	31.8	^bc^	644.5	±	9.2	^bc^	627.8	±	2.1	^bc^	522.4	±	18.1	^c^

^1.^ Monounsaturated fatty acids. ^2.^ Polyunsaturated fatty acids. ^3.^ Saturated fatty acid. Different superscript small letters in the same row for the same parameter denote a difference (*p* < 0.05) based on the Tukey test.

The objective was to evaluate the addition of germinated seeds to the crude extract of fenugreek seeds. The saturated fatty acid (SFA) contents were slightly lower for the extracts derived from germinated seeds (FGGW and FGGA) by 16 and 17%, respectively, compared to FGS. The PUFA contents were slightly higher at 9 and 8%, respectively, compared to FGS. Regarding the monounsaturated fatty acid (MUFA) contents, there was a 15% reduction in FGGW and FGGA compared to FGS. Shakuntala et al. (2011) [27] reported similar behavior in the germinated and non-germinated fractions of fenugreek seeds, specifically in the tegument and endosperm. The authors observed a reduction in the levels of unsaturated fatty acids from the non-germinated tegument (66%) to the germinated seed tegument (61%). Linolenic acid, which is present in large quantities in fenugreek seed extracts, exhibits vigorous antioxidant activity, serves as an antibacterial agent, and is helpful for disorders of the sexual and urinary systems [28]. The highest PUFA values (69% and 68.6%) are present in the extracts derived from individual seeds, FGGW, and FGGA.

#### 3.2.2. Free Fatty Acids in the Fenugreek Seed Extracts

The free fatty acids found in the sample from the breakdown of triglycerides by the extraction process are classified into categories: fatty acids, hydrocarbons, esters, tocopherols, monoglycerides, and phytosteroids (Table 3). Except for linoleic acid and cis-5,8,11-eicosatrienoic acid, the lipid profile of fenugreek seed extracts, raw or germinated, isolated or in mixtures, in different proportions remains the same. A total of 29 compounds were identified and classified according to specific groups, as shown in Table 3. The concentration of compounds in fenugreek extracts varied according to the type of seed (raw or germinated), the germination substrate (water or *Aloe vera*), and the mixtures of seeds in different proportions. Except for linoleic acid and cis-5,8,11-eicosatrienoic acid, the lipid profile of fenugreek seed extracts, raw or germinated, isolated or in mixtures, in different proportions remains the same.

#### 3.2.3. α-Tocopherol Content in Fenugreek Seed Extracts

Among the phytosteroids, α-tocopherol has the highest representation in fenugreek extracts, ranging from 99 to 296 mg per 100 g of extract (Table 2). The α-tocopherol (vitamin E) content found in the extracts obtained from germinated seeds and mixtures was up to 2.9 times higher than that found in FGS. Similar results have been obtained for extract from germinated flax seeds (*Linum usitatissimum* L.), in which germination promoted an increase in the levels of α-tocopherol [4].

Tocochromanols (tocopherols and tocotrienols) are vitamin E compounds that accumulate in angiosperm seeds and play an antioxidant role [29]. The methylated structure of α-tocopherol allows it to have the highest antioxidant activity among the tocopherols and be the most bioavailable [30]. Vitamin E has been shown to be effective in preventing diseases such as cancer [31], aging, arthritis, and cataracts due to its role in anti-inflammatory processes, its inhibition of platelet aggregation, its immune-boosting activity [31], its capacity to reduce lipid peroxidation and atherosclerotic lesions [32], and its action on LDL (low-density lipoprotein) during oxidation [33]. *Aloe vera*, which is used as a germination substrate for fenugreek seeds, is also an important source of α-tocopherol [34].

#### 3.2.4. Content of Phytosterols in Fenugreek Seed Extracts

Among the phytosterols identified, β-sitosterol emerges as the predominant compound, ranging from 522 to 683 mg 100 g^−1^ extract (Table 2). Compared to other extracts such as coconut, safflower, evening primrose [35], and linseed [4,35], fenugreek seeds display a similar sterol profile, with β-sitosterol predominating. Campesterol was also detected. Extraction using pressurized *n*-propane in a 2:1 FGS/GW ratio led to an increase of 26% in campesterol content (144.7 mg 100 g^−1^ extract) compared to the FGS (114.5 mg 100 g^−1^ extract).

The hydrocarbon squalene was present in the highest proportion (88 to 146 mg 100 g^−1^ extract) in fenugreek seed extracts extracted with pressurized *n*-propane (Table 2). There was an increase in the squalene content in FGGW and FGGA extracts compared to FGS extracts. Squalene, a key component of human sebum, exhibits notable biological activities, including antioxidant and hypolipidemic properties [36]. It is present in *Aloe Vera* leaves [37]. This may be a justification for the higher squalene content in FGGW (146.7 mg 100 g^−1^ extract) compared to all other extracts.

#### 3.2.5. Profile of Phenolic Compounds

The UHPLC-ESI-MS (Table 4) analysis identified 15 phenolic compounds in germinated fenugreek and raw seed extracts extracted using pressurized *n*-propane, including three flavonoid glycosides, kaempferol-dirhamnoside, apigenin-6,8-dipentoside, and apigenin-7-O-rutinoside, alongside phenolic acids such as caffeic acid and p-coumaric acid. The phenolic compounds that were reported in fenugreek seeds and were common to other studies were caffeic, p-coumaric, kaempferol, apigenin, and luteolin [38].

The phenolic compound profiles of raw and germinated fenugreek seed extracts, whether isolated or combined in various proportions, exhibited no significant differences. This finding aligns with the work of Gonda et al. 2023 [39], who investigated the concentrations of trigonelline and 4-hydroxyisoleucine in germinated fenugreek seeds over 72 h. Their results indicated that while germination induces substantial metabolic alterations in fenugreek, there are no pronounced qualitative shifts in phenolic compound levels compared to those in raw seeds.

Fenugreek seeds are rich in polyphenols and flavonoids, with proven antioxidant activity [40]. The flavonoids detected in the extracts consisted of quercetin, luteolin, apigenin, and kaempferol, which exhibited well-documented pharmacological properties. Kaempferol 3-O-glucoside, apigenin-7-O-rutinoside, and naringenin were also found in fenugreek ethyl acetate extract [38].

Naringenin was not identified in the extract profiles of raw or germinated fenugreek seeds, or in their mixtures extracted using pressurized *n*-propane.

Kaempferol reduces the risk of cardiovascular diseases and presents anticancer properties [41]. The kaempferol, apigenin, and luteolin present in fenugreek seeds reduced total cholesterol, triglyceride, and LDL levels and increased high-density lipoprotein concentrations in hypercholesterolemic rats [38]. Vitexin and isovitexin, identified as C-glycosylated flavones, display diverse pharmacological effects, including antioxidant, anti-inflammatory, antispasmodic, and anticancer activities [42]. The alkaloid trigonelline, present in fenugreek seeds, has antilipidemic properties, which may help control diabetes and hypercholesterolemia by improving insulin sensitivity [43].

Trigonelline and the amino acid 4-hydroxyisoleucine contribute to cognitive function enhancement, sugar absorption regulation, and insulin production modulation [44]. Quercetin demonstrates anti-inflammatory, antioxidant, anticancer, and cardiovascular protective activities [45]. It is also present in *Aloe vera* [9]. Polyphenols derived from *Aloe vera* may exhibit a range of antioxidant properties [9]. Luteolin exhibits antioxidant, anti-inflammatory, and tumor growth inhibitory properties [46]. Apigenin provides anxiolytic and neuroprotective effects in neurological disorders [46].

#### 3.2.6. Total Phenolic Compounds (TPCs)

The levels of TPCs ranged from 8.9 to 11.9 mg per 100 g of extract (Table 5). All the oils obtained with pressurized fluids showed similar TPC values (*p* < 0.05), suggesting that different pressures and solvent mixtures did not impact these parameters. Phenolic levels depend on extraction solvents [47].

The ultrasound-assisted extraction of fenugreek seeds germinated for 72 h demonstrated a decline in phenolic content relative to the ungerminated material [48]. Ethanol–water extraction of seed saponins at 75 °C yielded a TPC of 4.4 mg gallic acid equivalents (EAG) 100 g^−1^ [49]. Comparable TPC values have been reported for sunflower seed oil obtained by ultrasound-assisted extraction (11.34 mg EAG 100 g^−1^) [50]. The literature consistently indicates that extraction time, temperature, and botanical species substantially modulate phenolic yields [51] Accordingly, defining optimal germination parameters is essential to enhance the functional quality of sprouts [52]. The comparable phenolic levels observed between germinated and raw seeds in the present work suggest that the germination protocol adopted was adequate to preserve these compounds.

#### 3.2.7. β-Carotene Content

According to ANOVA, there was a difference in the β-carotene content between the extracts obtained from the raw seeds and the germinated seeds, as well as their mixes (*p* < 0.05). The β-carotene contents in FGWW and FGGA were 55% higher than those found in FGS, and 62% higher in FGS/GA 2:1 (Table 5). These values show that the germination process of fenugreek seeds, regardless of the substrate (water or *Aloe vera*), influenced the β-carotene content of fenugreek seed extracts.

Fenugreek extracts obtained from the raw and germinated seeds, as well as their mixtures, by pressurized *n*-propane extraction, showed high β-carotene contents (29 to 47 mg·100 g^−1^). Fenugreek seeds are known for their high β-carotene content [53]. β-carotene contents ranging from 19.00 to 24.64 mg·100 g^−1^ were found in fenugreek leaves [54]. *Aloe vera*, in addition to phenolic compounds and flavonoids, is a good source of β-carotene [46]. Thus, the high values obtained in fenugreek extracts obtained from seeds germinated in *Aloe vera* and its mixtures (44.6, 47.1, and 39.8 mg·100 g^−1^) may be associated with the addition of *Aloe vera* as a germination substrate.

Carotenoids are a group of plant pigments obtained by consuming a variety of fruits and vegetables [45]. Like α-tocopherol, carotenoids have protective antioxidant actions due to their mechanism of eliminating reactive oxygen species [55], such as singlet oxygen and other ROS [55,56], and protecting plasma lipoproteins from oxidation [45]. Carotenoids can act in the prevention of human diseases, such as liver damage, cancer, and cardiovascular diseases, or as photoprotectors [56]. Carotenoids can interact synergistically with other antioxidants [56], such as vitamin E (α-tocopherol). The protective effects of the combined oral supplementation of carotenoids and vitamin E have been shown to be efficient in protecting against erythema in humans and in reducing sensitivity to ultraviolet light [55].

β-Carotene is increasingly acknowledged as a significant modulator of type 2 diabetes mellitus (T2DM). Observational studies have demonstrated inverse correlations between circulating β-carotene levels and glycemic indices within diabetic populations. One study [57] identified a relationship wherein lower serum β-carotene concentrations were associated with reduced insulin sensitivity in individuals diagnosed with diabetes. Furthermore, a prospective analysis spanning a decade and involving approximately 37,000 participants revealed an inverse association between β-carotene intake and the incidence of T2DM, indicative of a potential protective effect [58]. Additional epidemiological data suggest that increased levels of β-carotene may positively influence outcomes related to metabolic syndrome [59].

#### 3.2.8. Radical Scavenging Activity (ABTS^•+^)

Radical scavenging capacity is a primary indicator of antioxidant potential. The ABTS^•+^ assay, widely used for this purpose, generates the ABTS^•+^ radical cation; its reduction by antioxidants leads to a spectrophotometrically detectable decrease in absorbance. Fenugreek extracts exhibited ABTS^•+^ scavenging activities between 3.0 and 6.2 µmol TE g^−1^ of extract. For comparison, fenugreek microgreens display an activity of 13.4 ± 0.0 µmol TE g^−1^ [3]. Accordingly, the value obtained for extracts from germinated fenugreek seeds (6.82 µmol TE g^−1^) lies within the expected range.

The antioxidant activity, measured by ABTS^•+^, of the extracts from germinated fenugreek seeds was twice as high (6.2 mmol of TE g^−1^ extract) when compared to FGS (3.0 mmol of TE g^−1^ extract). The extracts obtained from the mixture of germinated and non-germinated seeds also showed intermediate values (Table 5). Germination significantly increased the antioxidant capacity of soluble extracts in germinated seeds compared to raw seeds. This fact can be attributed to the increase in some antioxidant components in germinated seeds, such as antioxidant vitamins [3]. The high levels of α-tocopherol and β-carotene found in the FGGW and FGGA extracts are directly correlated with the highest values of antioxidant activity measured by ABTS^•+^. In amaranth, radish, and broccoli, the ABTS^•+^ antioxidant capacity was higher for germinated seeds compared to raw seeds [3].

The presence of polyphenols and flavonoids in fenugreek seeds can also act as a potent source of antioxidants [60]. Previous studies reported similar findings in chia, fenugreek, golden flaxseed, phacelia, and evening primrose seeds, demonstrating a positive correlation with total phenolic content [61]. Additionally, research indicates that the antioxidant efficacy of fenugreek extract varies based on the extraction method. Pressurized fluid extraction techniques obtained extracts with the highest antioxidant activity [61,62].

### 3.3. Principal Component Analysis

**Figure 1:**
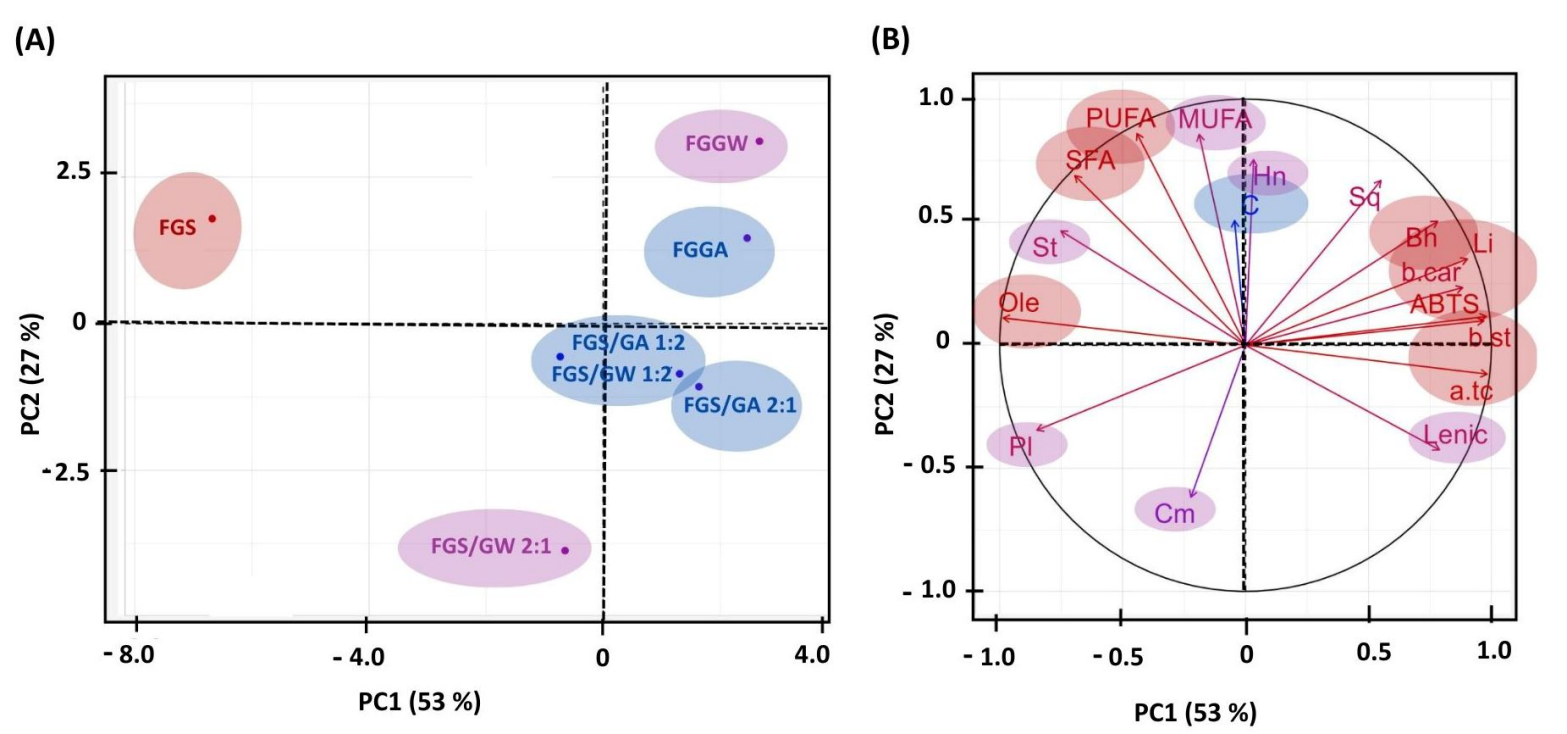
(**A**,**B**) Principal component analysis (PCA) relating to the first and second principal components (PC1 and PC2) relating to yield, ABTS^•+^, TPC, α-tocopherol, phytosterols, hydrocarbons, esters, fatty acids, and β-carotene, comparing the composition of raw and germinated fenugreek seeds. FGGW = fenugreek seed extract germinated in water; FGGA = fenugreek seed extract germinated in Aloe vera; FGS/GW 2:1 = extract from a mixture of raw fenugreek seeds and germinated seeds in water in a 2:1 ratio; FGS/GW 1:2 = extract from a mixture of raw fenugreek seeds and germinated seeds in water in a 1:2 ratio; FGS/GA 2 = extract from a mixture of raw fenugreek seeds and germinated seeds in *Aloe vera* in a 2:1 ratio; FGS/GA 1:2 = extract from a mixture of raw fenugreek seeds and germinated seeds in *Aloe vera* in a 1:2 ratio; FGS = raw fenugreek seed extract (ungerminated). Pl = palmitoleic acid; St = stearic acid; Ole = oleic acid; Hn = Heneicosanoic acid; Bh = Behenic acid; Li = Linoleic acid; Lenic = Linolenic acid; Sq = squalene; a.tc = α-Tocopherol; b.st = β-Sitosterol; b.car = β-carotene; ABTS^•+^ = radical monocation of 2,2’-azinobis-(3-ethylbenzothiazoline-6-sulfonic acid); Cm = Campesterol; Tt = trimethysilyl derivative; C = Total phenolic compounds; MUFA = monounsaturated fatty acid; PUFA= polyunsaturated fatty acid; SFA = saturated fatty acid.

The principal component analysis, which relates the contribution of each variable (ABTS^•+^, TPC, α-tocopherol, fatty acids, bioactive compounds, and β-carotene content) in relation to the composition of the extracts from raw and germinated fenugreek seeds (water and *Aloe vera*), as well as their combinations, explains 53% in PC1 and 27% in PC2, totaling 80% of the chemical composition of the extracts obtained from fenugreek seeds (Figure 1).

Fatty acids, antioxidant activity measured by ABTS^+^ (10.65%), oleic acid (10.78%), α-tocopherol (10.71%), and β-sitosterol (10.42%) were identified as the primary contributors to the chemical composition of fenugreek extracts, irrespective of the seed type (raw, germinated, or blended). In contrast, the lowest contributions were attributed to heneicosanoic acid (0.01%) and total phenolic content (0.02%). A substantial contribution to the chemical composition of fenugreek extracts was provided by FGS, accounting for 71% of the total variance explained in PC1. The total phenolic content (C) in extracts obtained from fenugreek seeds germinated in *Aloe vera* and its respective mixtures were identified as the most significant among the evaluated samples. Seeds germinated at 25 °C for 72 h also demonstrated elevated levels of phenolic compounds and antioxidant activity [8]. Similar increases in phenolic content, relative to raw seeds, have been reported for germinated seeds of *Sinapis arvensis* L. [5] and *Fagopyrum tataricum* [63].

PC2 allows the differentiation of extracts based on their chemical contributions. Extracts exhibiting the highest compositional contributions were located on the negative axis, with FGS positioned on the left side of the graph, while FGGW (11%) and FGGA (10%) were located on the right. On the positive axis, extracts with the lowest compositional contributions, namely FGS/GA 2:1, FGS/GA 1:2, FGS/GW 2:1, and FGS/GW 1:2, were positioned. On the positive axis, the major contributors to the chemical profile included saturated fatty acids (SFAs), MUFAs, PUFAs, ABTS^+^ activity, β-carotene, and β-sitosterol, particularly in FGGW, FGGA, and FGS extracts. Conversely, on the negative axis, linolenic acid and α-tocopherol were identified as the predominant contributors in extracts derived from seed mixtures (FGS/GA 1:2, FGS/GA 2:1, FGS/GW 2:1, and FGS/GW 1:2).

### 3.4. Increases in Bioactive Compounds Through the Germination Process and the Use of an Aloe Vera Elicitor

After germination, significant changes were observed, with a marked increase in PUFAs (9.5%) and a corresponding decrease in oleic acid content (15%). In plant seed extracts, fatty acids play an important role in germination, since lipids are the main reservoir and consequent source of energy for the developing embryos [64]. In oilseed germination, lipids are an essential source of energy and carbon for the development of the embryo [65]. Fenugreek seeds predominantly contain unsaturated fatty acids [66]. This is why there is a decrease in the content of oleic acid (a monounsaturated fatty acid) in germinated seeds; some of these fatty acids are utilized as an energy source during the germination process. The low content of MUFAs in the extract from germinated fenugreek seeds indicates that oleic acid is preferentially utilized for energy production or as a substrate for synthesizing other compounds during germination.

This change in fatty acid composition suggests a metabolic response during germination by mobilizing stored reserves (fatty acids) to supply nutrients to the plant until it can produce its own food via photosynthesis [67]. The germination of fenugreek seeds improved the lipid profile and quality of the extracts obtained, promoting extracts with higher levels of unsaturated fatty acids and lower levels of SFAs. This information supports germination as an effective seed pretreatment process for improving the nutritional properties of extracts at a low cost. FGGW and FGGA show potential as rich sources of unsaturated acids for health-promoting applications. The high PUFA concentration in fenugreek extract yields promising results when extracted using pressurized *n*-propane, facilitating the recovery of bioactive compounds with significant health benefits.

Biotic elicitor substances, such as the *Aloe vera* used in this study, induce physiological changes in the plant. In response to this stress, a series of defense mechanisms are activated on the surface of the plasma membrane, increasing the synthesis of phytochemicals [68]. *Aloe vera* gel (AVG) showed positive effects on the germination of *Dianthus barbatus* [69] and *Cicer arietinum* L. (chickpea) [70]. *Aloe vera* extract (ALE) was successfully used on the growth, multiplication, and phytochemical composition of *Lavandula officinalis* seedlings in vitro, affecting the composition of some fatty acids (palmitic and stearic) [71]. Incorporating *Aloe vera* into seed treatments can introduce various compounds such as phytohormones, vitamins, amino acids, and enzymes [70]. Regarding germination processes, *Aloe vera* can act by breaking the dormancy barrier, leading to greater germination [72]. The limitations of this study are related to elucidating the physiological mechanisms involved in increasing the synthesis of bioactive compounds promoted by the germination process and the elicitation with *Aloe vera* in fenugreek extracts using *n*-propane.

### 3.5. In Vitro Estimation of α-Amylase and α-Glucosidase Inhibitory Activity

The conventional approach to managing type II diabetes mellitus involves inhibiting starch-digesting enzymes, such as α-amylase, to reduce carbohydrate digestion and control glycemic responses. While antidiabetic medications are effective, they can lead to side effects such as abdominal discomfort and diarrhea, prompting interest in natural alternatives. Bioactive compounds from natural sources that inhibit α-amylase and α-glucosidase are promising for mitigating these adverse effects. Table 6 shows the inhibitory percentages of these enzymes by fenugreek sample.

The extracts showed significant differences in inhibitory capacity (*p* < 0.05). Among them, for α-amylase, the FGS/GW 1:2 (95%) and FGS (92%) extracts showed the highest inhibition rate. Regarding the inhibition of α-glucosidase, which is essential for reducing carbohydrate absorption and controlling postprandial blood glucose levels, the FGS and FGGW extracts showed the highest inhibitory activity, reaching values of 99% and 96%, respectively. The combination of seeds and germinated seeds also showed significant inhibitory activity (>65%) (*p* < 0.05).

The findings presented here exceed those reported by Shawky et al. 2022 [73], which showed a 52.7% inhibition of α-amylase and a 70% inhibition of α-glucosidase in fenugreek leaf extracts. These results indicate that germinated fenugreek seeds have significant natural antidiabetic potential for controlling hyperglycemic responses in diabetic patients. Fenugreek seeds contain galactomannan, a soluble fiber that makes up approximately 45% to 60% of their composition [40]. This fiber can help reduce postprandial glycemia by delaying the intestinal absorption of carbohydrates [40].

Other components present in fenugreek seeds, such as trigonelline, have potential antidiabetic activity [74]. The FGS/GA 2:1 and FGS/GA 1:2 extracts presented the second highest inhibition value of α-glucosidase and α-amylase, indicating that there may have been the translocation of phenolic compounds and other compounds with antidiabetic actions from the *Aloe vera* gel. *Aloe vera* leaf pulp extract was effective in reducing blood sugar [75]. Aqueous extracts of fenugreek exhibit potential as an adjunctive therapeutic agent for diabetes management, allowing for significant dosage reductions of conventional pharmaceutical interventions [40]. Hydroalcoholic extracts of fenugreek seeds reduced memory impairments resulting from diabetes [76].

### 3.6. In Vitro Evaluation of Anticancer Activity

The extract from the mixture of germinated fenugreek seeds in *Aloe vera* and raw seeds (FGS/GA 2:1 and FGS/GA 1:2) under different raw seed/germinated seed ratios (Table 7) were evaluated for their in vitro cytotoxic effects on the HeLa and SiHa cancer cell lines.

Owing to the high operational costs associated with assay development, only those extracts derived from mixtures of raw and germinated seeds that exhibited the most outstanding TPCs were selected. This selection criterion aligns with the primary objective of assessing the impact of germination on the bioactive profile of *n*-propane-extracted fenugreek fractions. MTT cell viability assays (Table 6) demonstrated marked cytotoxicity for the FGS/GA 2:1 and FGS/GA 1:2 extracts, with substantial growth inhibition after 24 h, as reflected by their IC_50_ values. The most pronounced antiproliferative response was observed in HeLa cells, which exhibited the lowest IC_50_ values for both extracts (107.7 and 104.7 µg·mL^−1^).

Preliminary phytochemical profiling of the extracts was conducted using gas chromatography–mass spectrometry (GC–MS), revealing the presence of diverse bioactive secondary metabolites. Extracts derived from germinated seeds were notably enriched in trigonelline and apigenin compounds with well-documented anticancer and antidiabetic activities [77]. Both metabolites have been shown to enhance the susceptibility of malignant cells to apoptosis [44,78], and dietary supplementation with fenugreek extracts has been correlated with a reduced incidence of colon carcinogenesis [79]. Concurrently, *Aloe vera* has demonstrated therapeutic efficacy against more than 57 cancer types, including gastric and colorectal malignancies [80]. In agreement with these observations, the FGS/GA 2:1 and FGS/GA 1:2 formulations displayed pronounced antitumor activity, concomitant with elevated apoptotic indices in treated cells. These results are consistent with prior reports attributing such effects to a down-regulation of proteasome expression and activity within cancer cells [44].

## 4. Conclusions

Increases in vitro antioxidant, anti-hyperglycemic, and anticancer properties, an improved lipid profile due to an increase in PUFAs and decreases in SFAs, α-tocopherol content (by approximately three-fold), β-sitosterol, and squalene, and bioactive compounds with nutritional and therapeutic relevance were identified in the extracts obtained from the germination of fenugreek in water and *Aloe vera*. Phenolic compounds with well-documented pharmacological activities, such as quercetin, luteolin, apigenin, and kaempferol, were identified in all fenugreek extracts. These results demonstrated the potential of germination and elicitation with *Aloe vera* in enriching fenugreek seed extracts extracted with pressurized *n*-propane. However, the causal links between the biological activity of germination and elicitation with *Aloe vera* are not firmly established and should be evaluated in further studies. FGS/GA 2:1 and 1:2 showed potential anticancer activity in vitro in cervical cancer (HeLa) and colon cancer (SiHa) cell lines. These results suggest that these modified sprouts provide health benefits. However, the findings emphasize the need for additional clinical studies to consolidate their therapeutic efficacy. TPC values were higher for extracts obtained via pressurized *n*-propane than for those obtained via conventional extraction. The specialized open literature provides several examples that demonstrate the scalability of the extraction process using pressurized fluids as an economically viable process for industrial applications. This process offers several advantages, including reduced solvent consumption, shorter extraction times, the elimination of post-processing steps, and the production of high-quality products that do not contain solvents. These characteristics, which are intrinsic to the processing of natural products using pressurized fluids, suggest the potential to increase the health benefits of the products.

## Figures and Tables

**Table 1:** Mass percentage yield of FGS, FGGW, FGGA, FGS/GW, and FGS/GA in different proportions (1:2 and 2:1), obtained by pressurized *n*-propane (PRO).

Description of Extracts	Sample Codes	Yield (%)		CV (%)
Fenugreek seed extract germinated in water	FGGW	3.1 ± 0.8	^a^	25.9
Fenugreek seed extract germinated in *Aloe vera*	FGGA	3.2 ± 0.1	^a^	4.2
Extract from a mixture of raw fenugreek seeds and germinated seeds in water in a 2:1 ratio	FGS/GW 2:1	4.4 ± 0.4	^a^	9.3
Extract from a mixture of raw fenugreek seeds and germinated seeds in water in a 1:2 ratio	FGS/GW 1:2	4.3 ± 0.4	^a^	10.2
Extract from a mixture of raw fenugreek seeds and germinated seeds in *Aloe vera* in a 2:1 ratio	FGS/GA 2:1	3.8 ± 0.5	^a^	15.0
Extract from a mixture of raw fenugreek seeds and germinated seeds in *Aloe vera* in a 1:2 ratio	FGS/GA 1:2	3.7 ± 0.2	^a^	4.4
Raw fenugreek seed extract (ungerminated)	FGS	4.6 ± 0.7	^a^	15.4

CV: coefficient of variation; different lowercase letters and superscripts in the same column denote differences (*p* < 0.05) based on Tukey’s test.

**Table 3:** Composition of free fatty acids, phytosteroids, hydrocarbons, and olein monoglycerides measured in relative percentage area present in raw fenugreek seeds extract (FGS), germinated fenugreek seed extract (FGGW and FGGA), and mixed extracts (FGS/GW and FGS/GA) in different proportions (1:2 and 2:1), obtained by pressurized *n*-propane.

		FGGW	FGGA	FGS/GW 2:1	FGS/GW 1:2	FGS/GA 2:1	FGS/GA 1:2	FGS
Classes	Funcion	Relative area (%)						
Hexanoic acid	acid	0.1	0.1	0.2	0.2	0.4	0.4	0.8
Hexadecanoic acid	acid	9.4	9.4	14.3	10.8	11.9	9.5	14.7
Heptadecanoic acid	acid	0.3	0.2	0.4	0.3	0.4	0.2	0.4
9,12-Octadecadienoic acid (Z,Z) (**ω-**6 fatty-acid)	acid	33.8	33.6	30.2	32.2	29.3	33.1	33.3
Oleic acid (ω-3 fatty-acid)	acid	34.7	34.4	29.7	32.4	29.0	33.8	31.6
Linolenic acid	acid	2.2	1.9	3.4	2.6	3.6	2.6	1.8
Octadecanoic acid	acid	5.0	5.3	8.7	6.0	6.9	6.2	6.7
cis-5,8,11-Eicosatrienoic acid	acid	0.5	0.3	0.1	0.3	0.0	0.3	0.6
Eicosanoic acidr (ω-6 fatty-acid)	acid	1.4	1.4	1.7	1.5	2.0	1.3	1.6
Heneicosanoic acid	acid	0.3	0.2	0.2	0.2	0.2	0.2	0.3
Docosanoic acid	acid	2.6	3.0	1.8	2.2	2.0	2.0	2.2
Tricosanoic acid	acid	0.7	0.7	0.7	0.8	0.8	0.7	0.5
Tetracosanoic acid	acid	2.4	2.4	2.4	3.0	3.3	2.2	1.2
Hexacosanoic acid	acid	2.4	3.9	5.3	4.4	5.0	3.9	1.3
Linoleic acid ethyl ester (ω-6 fatty-acid)	esther	0.02	0.0	0.00	0.02	0.0	0.00	0.4
9-Octadecenoic acid, 2-[(trimethylsilyl)oxy]-1-[[(trimethylsilyl)oxy]methyl]ethyl ester	monoglycerides	3.7	2.6	0.7	2.7	0.5	3.2	2.2
Octadecanoic acid, 2,3-bis[(trimethylsilyl)oxy]propyl ester	monoglycerides	0.5	0.6	0.2	0.5	0.5	0.6	0.4
MUFA ^1^		5.9	4.5	4.1	5.2	4.1	5.8	4.0
PUFA ^2^		71.1	70.4	62.4	67.1	61.1	69.1	68.0
SFA ^3^		23.0	25.1	33.5	27.6	30.6	25.1	28.0
Saturated		23.0	25.1	33.5	27.6	30.6	25.1	28.0
Insaturated		77.0	74.9	66.5	72.4	65.2	74.9	72.0
1-Monopalmitin	monoglycerides	0.0	0.2	0.1	0.1	0.2	0.0	17.5
1-Monooleoylglycerol	monoglycerides	0.2	0.6	0.2	0.5	0.6	0.5	0.4
Squalene	hydrocarbon	14.7	12.6	17.7	15.2	19.1	22.2	14.5
Nonacosane	hydrocarbon	23.4	28.6	25.2	27.3	17.9	22.5	22.2
Hentriacontane	hydrocarbon	61.9	58.8	57.2	57.5	63.0	55.3	63.3
a-Tocopherol	tocopherol	11.1	8.3	13.2	10.9	11.5	8.8	0.7
Cholesterol	phytosteroids	6.0	5.9	7.5	7.5	5.0	5.2	7.5
Campesterol	phytosteroids	19.5	17.2	6.2	18.2	14.5	15.6	14.2
β-Sitosterol	phytosteroids	48.8	54.2	52.3	46.9	53.1	53.0	55.4
Lupenyl acetate	phytosteroids	14.6	14.5	20.8	16.5	15.9	17.4	22.3

^1.^ Monounsaturated fatty acids. ^2.^ Polyunsaturated fatty acids. ^3.^ Saturated fatty acid.

**Table 4:** Qualitative analysis of FGS, FGGW, FGGA, FGS/GW, and FGS/GA extracts in different proportions (1:2 and 2:1) using LC-MS, obtained using pressurized *n*-propane.

Identification	Molecular Weight	MS (±)	Samples (Intensity Values)						
			FGGW	FGGA	FGS/GW 2:1	FGS/GW 1:2	FGS/GA 2:1	FGS/GA 1:2	FGS
Caffeoyl glucose	342	341	1.4 × 10^6^	5.5 × 10^6^	7.5 × 10^6^	1.0 × 10^7^	1.6 × 10^7^	1.2 × 10^7^	7.2 × 10^6^
*p*-coumaric acid derivative	164	311	8.7 × 10^6^	2.9 × 10^7^	6.1 × 10^6^	2.3 × 10^7^	9.0 × 10^6^	3.6 × 10^7^	3.3 × 10^7^
Caffeic acid derivative	180	377	5.0 × 10^5^	1.9 × 10^6^	1.7 × 10^6^	3.8 × 10^6^	5.2 × 10^6^	3.0 × 10^6^	4.1 × 10^6^
Vitexin	432	433	2.8 × 10^5^	9.8 × 10^5^	2.4 × 10^6^	2.8 × 10^6^	7.4 × 10^5^	5.4 × 10^6^	1.7 × 10^6^
Isovitexin	432	433	2.8 × 10^5^	9.8 × 10^5^	2.4 × 10^6^	2.8 × 10^6^	7.4 × 10^5^	5.4 × 10^6^	1.7 × 10^6^
Kaempferol-dirhamnoside	741	593	6.2 × 10^5^	1.2 × 10^6^	2.8 × 10^6^	1.3 × 10^6^	2.8 × 10^5^	7.7 × 10^5^	1.6 × 10^6^
Apigenin-6,8 dipentoside	534	533	4.3 × 10^5^	2.2 × 10^6^	1.7 × 10^6^	1.5 × 10^6^	1.8 × 10^6^	1.8 × 10^6^	1.6 × 10^6^
Kaempferolrhamnoside	432	432	5.3 × 10^5^	2.1 × 10^6^	1.8 × 10^6^	8.0 × 10^6^	3.1 × 10^6^	1.5 × 10^6^	1.1 × 10^6^
Apigenin-7-O-rutinoside	578	579	5.1 × 10^4^	7.7 × 10^5^	1.9 × 10^6^	5.6 × 10^5^	6.8 × 10^5^	3.9 × 10^5^	1.6 × 10^6^
Quercetin	302	301	2.5 × 10^6^	2.9 × 10^6^	6.0 × 10^6^	6.2 × 10^6^	4.7 × 10^6^	4.9 × 10^6^	2.0 × 10^6^
Luteolin	286	288	4.6 × 10^5^	6.2 × 10^5^	1.7 × 10^5^	8.2 × 10^4^	1.0 × 10^5^	9.1 × 10^5^	6.6 × 10^5^
Apigenin	270	271	2.8 × 10^6^	7.5 × 10^6^	7.9 × 10^5^	6.1 × 10^6^	4.7 × 10^6^	1.1 × 10^7^	4.4 × 10^6^
Caffeic acid	180	179	2.5 × 10^6^	8.0 × 10^5^	4.5 × 10^5^	2.5 × 10^6^	2.1 × 10^6^	6.3 ∙ 10^5^	3.7 × 10^6^
Trigonelline	137	-	3.1 × 10^5^	6.8 × 10^4^	5.6 × 10^4^	2.4 × 10^5^	ND	1.8 × 10^5^	2.4 × 10^5^
4-hydroxyisoleucine	147	130	6.4 × 10^5^	1.2 × 10^6^	2.8 × 10^5^	1.2 × 10^6^	1.1 × 10^6^	4.2 × 10^5^	1.3 × 10^6^

**Table 5:** Content of minority compounds, total phenolic components, antioxidant capacity, and β-carotene in FGS, FGGW and FGGA, and FGS/GW and FGS/GA extracts in different proportions (1:2 and 2:1), obtained using pressurized *n*-propane.

Sample	TPC				ABTS				β-Carotene			
	mg EAG 100 g^−1^ Extract				µmol de TE g^−1^ Extract				mg 100 g ^−1^ Extract			
FGGW	10.2	±	1.5	^a^	6	±	0.2	^a^	45.1	±	0.6	^ab^
FGGA	11.9	±	0.7	^a^	6	±	0.2	^a^	44.6	±	1.0	^ab^
FGS/GW 2:1	8.9	±	0.9	^a^	5	±	0.04	^c^	32.4	±	0.6	^c^
FGS/GW 1:2	9.9	±	0.9	^a^	6	±	0.07	^ab^	43.5	±	4.0	^ab^
FGS/GA 2:1	11.5	±	0.3	^a^	6	±	0.3	^ab^	47.1	±	0.6	^a^
FGS/GA 1:2	11.3	±	0.8	^a^	5	±	0.2	^bc^	39.8	±	0.3	^b^
FGS	11.2	±	2.6	^a^	3	±	0.3	^d^	29.2	±	0.8	^c^

Different lowercase letters superscript in the same column for the same parameter denote a difference (*p* < 0.05) based on Tukey’s test.

**Table 6:** Inhibition data for in vitro testing of the enzymes α-glucosidase and α-amylase in FGS, FGGW and FGGA, and FGS/GW and FGS/GA extracts in different proportions (1:2 and 2:1), obtained using pressurized *n*-propane.

Sample	α-Glucosidase				α-Amylase			
FGGW	95.5	±	1.0	^a^	27.7	±	2.7	^d^
FGGA	53.4	±	3.2	^d^	52.7	±	2.7	^c^
FGS/GW 2:1	65.1	±	3.8	^c^	15.2	±	0.6	^e^
FGS/GW 1:2	68.2	±	1.2	^c^	95	±	3.5	^a^
FGS/GA 2:1	82.4	±	0.9	^b^	75.9	±	2.6	^b^
FGS/GA 1:2	66.2	±	1.0	^c^	72.6	±	2.0	^b^
FGS	98.9	±	1.4	^a^	91.5	±	6.6	^a^

Different lowercase letters in superscript in the same column for the same parameter denote a difference (*p* < 0.05) based on Tukey’s test.

**Table 7:** Half-maximum inhibitory concentrations (IC_50_) of the extract’s mixture (germinated fenugreek seeds) in *Aloe vera* samples against HeLa and Siha cells.

Samples	HeLa (μg/mL)	SiHa (μg/mL)
FGS/GA 2:1	107.7	188.5
FGS/GA 1:2	104.7	202.5

IC_50_: half-maximum inhibitory concentration.

## Data Availability

The original contributions presented in this study are included in the article. Further inquiries can be directed to the corresponding author(s).

## Abbreviations

The following abbreviations are used in this manuscript:

FGGW	Fenugreek seed extract germinated in water
FGGA	Fenugreek seed extract germinated in *Aloe vera*
FGS/GW 2:1	Extract from a mixture of raw fenugreek seeds and germinated seeds in water in a 2:1 ratio
FGS/GW 1:2	Extract from a mixture of raw fenugreek seeds and germinated seeds in water in a 1:2 ratio
FGS/GA 2:1	Extract from a mixture of raw fenugreek seeds and germinated seeds in *Aloe vera* in a 2:1 ratio
FGS/GA 1:2	Extract from a mixture of raw fenugreek seeds and germinated seeds in *Aloe vera* in a 1:2 ratio
FGS	Raw fenugreek seed extract (ungerminated)
TPCs	Total phenolic compounds
ABTS	2,2′-Azinobis-(3-Ethylbenzthiazolin-6-Sulfonic Acid
PUFA	Polyunsaturated fatty acid
MUFA	Monounsaturated fatty acid
SFA	Saturated fatty acid
